# Stability Study of Flexible 6,13-Bis(triisopropylsilylethynyl)pentacene Thin-Film Transistors with a Cross-Linked Poly(4-vinylphenol)/Yttrium Oxide Nanocomposite Gate Insulator

**DOI:** 10.3390/polym8030088

**Published:** 2016-03-16

**Authors:** Jin-Hyuk Kwon, Xue Zhang, Shang Hao Piao, Hyoung Jin Choi, Jin-Hyuk Bae, Jaehoon Park

**Affiliations:** 1 School of Electronics Engineering, Kyungpook National University, Daegu 41566, Korea; rnjs3055@naver.com; 2 Department of Electronic Engineering, Hallym University, Chuncheon 24252, Korea; zhangxue00@naver.com; 3 Department of Polymer Science and Engineering, Inha University, Incheon 22212, Korea; sanghoo1105@gmail.com

**Keywords:** flexible electronics, transistor, polymeric insulator, nanocomposite, stability

## Abstract

We investigated the electrical and mechanical stability of flexible 6,13-bis(triisopropylsilylehtynyl)pentacene (TIPS-pentacene) thin-film transistors (TFTs) that were fabricated on polyimide (PI) substrates using cross-linked poly(4-vinylphenol) (c-PVP) and c-PVP/yttrium oxide (Y_2_O_3_) nanocomposite films as gate insulators. Compared with the electrical characteristics of TIPS-pentacene TFTs with c-PVP insulators, the TFTs with c-PVP/Y_2_O_3_ nanocomposite insulators exhibited enhancements in the drain current and the threshold voltage due to an increase in the dielectric capacitance. In electrical stability experiments, a gradual decrease in the drain current and a negative shift in the threshold voltage occurred during prolonged bias stress tests, but these characteristic variations were comparable for both types of TFT. On the other hand, the results of mechanical bending tests showed that the characteristic degradation of the TIPS-pentacene TFTs with c-PVP/Y_2_O_3_ nanocomposite insulators was more critical than that of the TFTs with c-PVP insulators. In this study, the detrimental effect of the nanocomposite insulator on the mechanical stability of flexible TIPS-pentacene TFTs was found to be caused by physical adhesion of TIPS-pentacene molecules onto the rough surfaces of the c-PVP/Y_2_O_3_ nanocomposite insulator. These results indicate that the dielectric and morphological properties of polymeric nanocomposite insulators are significant when considering practical applications of flexible electronics operated at low voltages.

## 1. Introduction

Over the last few decades, organic thin-film transistors (TFTs) have been extensively studied as candidates for next-generation electronics, because of their simple and large-area applicable processing, low manufacturing cost, light weight, and mechanical flexibility [1,2,3]. In particular, the pliability of organic materials facilitates mechanical compatibility between organic TFTs and plastic substrates, thereby giving organic TFTs potential applicability in bendable and rollable electronic products. In addition, since some organic materials can be solution-processed at a relatively low temperature, solution-processes, such as ink-jet and screen printing, have been considered in order to maximize several unique advantages of organic TFTs. As a result, organic TFTs are expected to be used in diverse applications, such as flexible displays, RFID tags, and various sensors [4,5,6].

Nevertheless, the relatively high operating voltage of organic TFTs, which comes from their use of low dielectric polymer gate insulators, has been regarded as an impediment to their commercialization. Ceramic-based inorganic materials with a high dielectric constant (high-*k*) can be used as a way to lower the operating voltage of organic TFTs [7,8,9]. However, most high-*k* materials that are suitable for organic TFTs are processed via deposition techniques and sol-gel methods and require high processing temperatures that are not suitable for plastic substrate application. In order to solve this problem, some research groups have devised the use of nanocomposite insulators that can be formed by annealing high-*k* nanoparticle blended polymer solutions at a relatively low temperature [10,11,12]. However, a study on the mechanical and electrical stability of organic TFTs with nanocomposite insulators built on plastic substrates has not yet been reported, while device reliability should also be an important consideration in their further application and development.

In this work, we fabricated 6,13-bis(triisopropylsilylehtynyl)pentacene (TIPS-pentacene TFTs with two types of gate insulators on polyimide plastic substrates. Here, yttrium oxide (Y_2_O_3_) nanoparticles were used to form the nanocomposite gate insulator, which exhibit a relatively wide band gap energy of 6.0 eV compared to that (3.2 eV) of the commonly used TiO_2_ nanoparticles for nanocomposite gate insulators; the wide band gap feature of Y_2_O_3_ is very advantageous on aspects of illumination stability of TFTs [13]. The first type of device used cross-linked poly(40vinylphenol)(c-PVP)/Y_2_O_3_ nanocomposite gate insulators, and the second type used normal c-PVP gate insulators. The electrical stability of these devices was then analyzed by measuring the time variations of the saturation drain current, the gate leakage current, and the threshold voltage shift under fixed bias conditions. In addition, the mechanical stability of the devices was analyzed by measuring the variations in the output current and the transfer characteristic curve under repetitive bending. Consequently, we were able to evaluate the mechanical and electrical stability of the TIPS-pentacene organic TFTs using the nanocomposite insulator.

## 2. Materials and Methods

**Figure 1:**
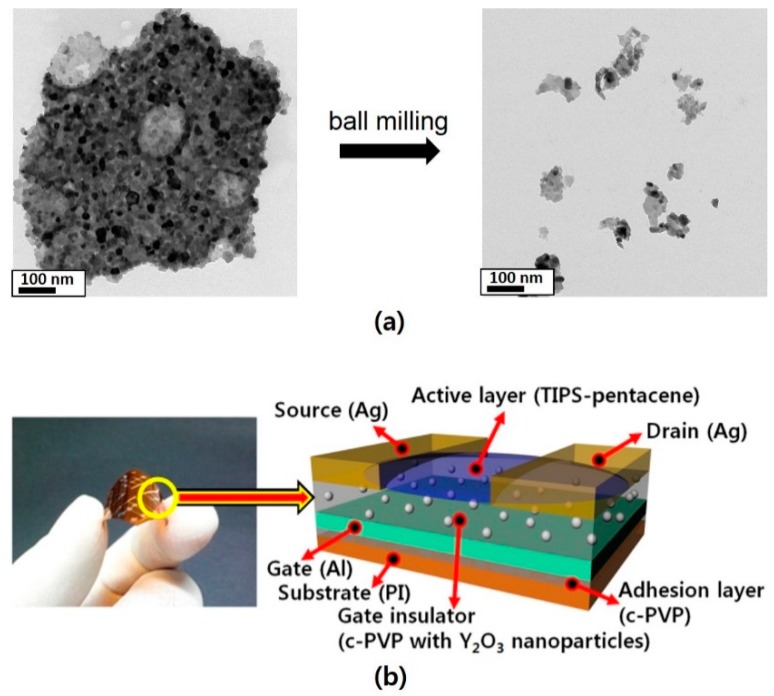
(**a**) TEM images of Y_2_O_3_ particles; (**b**) Schematic of the fabricated flexible TIPS-pentacene TFT.

For this study, we used bottom-gate and top-contact structured TIPS-pentacene TFTs with c-PVP/Y_2_O_3_ nanocomposite gate insulators, and with normal c-PVP gate insulators, both built on PI substrates. To form a solution of cross-linkable PVP, PVP and a cross-linking agent (methylated poly(melamine-co-formaldehyde), MMF) were dissolved in propylene glycol methyl ether acetate (PGMEA). The percentage of PVP dissolved in the PGMEA was 15 wt %, and the PVP:MMF mass ratio was 1:1. The resulting solution was used for the normal c-PVP insulator. To form the Y_2_O_3_ nanocomposite solution, glass beads and Y_2_O_3_ nanoparticles were mixed with the PVP solution, and the composite solution was subsequently ball milled to break the aggregates of Y_2_O_3_ nanoparticles. In our experiments, the weight fraction of Y_2_O_3_ nanoparticles was fixed at 1.5 wt % in the nanocomposite insulator. The transmission electron microscope (TEM) images in Figure 1a show that the cluster sizes of the Y_2_O_3_ particles were significantly reduced by the ball milling. It should be noted that the elimination of such aggregates is essential in order to ensure the uniform dispersion of the Y_2_O_3_ particles in the composite solution. Prior to TFT fabrication, the PI substrates were cleaned with acetone, 2-propanol, and deionized water in sequence for 10 min each. To form the adhesion layer for the aluminum (Al) gate electrodes, the PVP solution was spin-coated at 2000 rpm for 30 s onto the cleaned PI substrates, and then baked on a hot plate at 180 °C for 1 h to induce cross-linking between the PVP chains. For the gate electrodes, 38-nm-thick Al was thermally deposited at a rate of 0.1 nm/s onto the c-PVP-coated substrate through a shadow mask. For the gate insulators, the PVP solution with or without Y_2_O_3_ nanocomposite was spin-coated at 2000 rpm for 30 s onto the substrates with the deposited Al gate electrodes, and then baked on a hot plate at 180 °C for 1 h. Before formation of the active layers, it should be noted that there is no surface treatments to induce any aligned features in active layer molecules. For the active layers, a solution of TIPS-pentacene dissolved in anisole at 2 wt % was drop casted onto the gate insulators, and then baked on a hotplate at 60 °C for 30 min. Finally, 90-nm-thick silver (Ag) was thermally deposited at a rate of 0.2 nm/s onto the substrates through another mask for source/drain electrodes, of which the channel width and length was 1000 and 100 μm, respectively. A photograph and a schematic cross-sectional view of our device are shown in Figure 1b.

The surface morphology of films was studied using an atomic force microscope (AFM) (Nanoscope IVa, Coventry, UK), and the dielectric property of the gate insulators was measured using an impedance analyzer (HP 4192A, Santa Clara, CL, USA). The electrical characteristics of the TFTs were measured using a semiconductor analyzer (EL 421C, Seoul, Korea).

## 3. Results and Discussion

**Figure 2:**
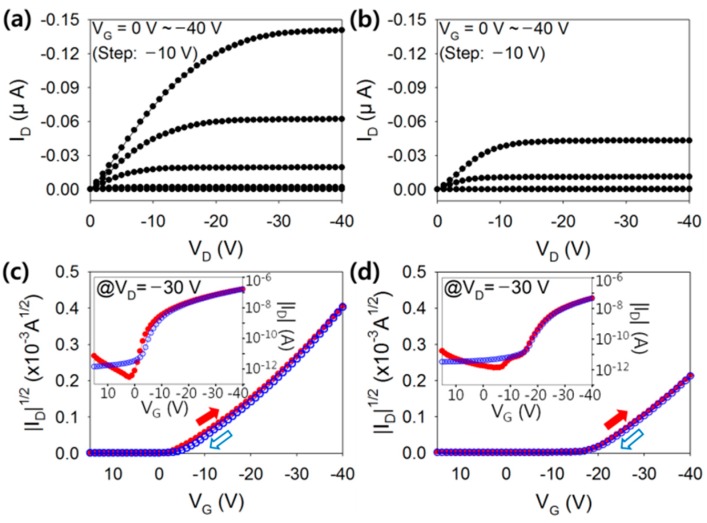
Output characteristics of the flexible TIPS-pentacene TFTs with the (**a**) c-PVP/Y_2_O_3_ composite and (**b**) c-PVP gate insulators |*I*_D_| *vs. V*_G_ and |*I*_D_|^1/2^ *vs. V*_G_ plots of TIPS-pentacene TFTs with the (**c**) c-PVP/Y_2_O_3_ composite and (**d**) c-PVP gate insulators.

Figure 2 shows the output and transfer characteristics obtained from the fabricated TIPS-pentacene TFTs with the c-PVP/Y_2_O_3_ nanocomposite gate insulator (denoted as Type A device), and those with the typical c-PVP gate insulator (denoted as Type B device). The output characteristic was measured by changing the drain voltage from 0 to −40 V in increments of −1 V at different gate voltages. Note that the drain current, as shown in Figure 2a,b, is found to be sufficiently saturated when the drain voltage reaches −30 V for the various gate bias conditions. Thus, the corresponding transfer characteristic was measured by changing the gate voltage from 15 to −40 V in increments of −1 V at a constant drain voltage of −30 V. As shown in Figure 2a,b, the output drain current of Type A device was higher than that of Type Be device, while both devices exhibited good current saturation behavior. The saturation currents of Type A device and Type B device were −0.141 and −0.0432 μA, respectively, at the drain and gate voltage of −40 V. As shown in Figure 2c,d, the on-state drain current of Type A device was higher than that of Type B device. The on-state drain current of Type A device was −0.165 μA and that of Type B device was −0.0462 μA at a gate voltage of −40 V and a drain voltage of −30 V. The higher drain current of Type A device in the output and transfer characteristics was attributed to the higher dielectric capacitance of the nanocomposite gate insulators. Note that the measured capacitances of the normal c-PVP insulator and the c-PVP/Y_2_O_3_ nanocomposite insulator were 118.8 pF/cm^2^ and 198.8 pF/cm^2^, respectively. The increase in the capacitance of the c-PVP/Y_2_O_3_ nanocomposite insulator can be ascribed to the high-k property of Y_2_O_3_ (approximately 16) [14]. In addition, a considerable reduction in the threshold voltage was observed in Type A device. The threshold voltage of Type A device was −5 ± 1 V, and that of Type B device was –20 ± 3 V. This indicated that the formation of TFT channels could be significantly improved through the adoption of high-dielectric-capacitance nanocomposite insulators; a higher dielectric capacitance contributes to accumulating more charge carriers in the conducting channel of TFTs, thereby reducing the threshold voltage. In hysteresis tests, no significant variation was observed in the current level when the gate voltage was swept up and down for both Type A and Type B devices. Note that the hysteresis behavior is directly related to the stable operation of device. From the results shown in Figure 2, it was confirmed that Type A device operated properly and showed enhanced electrical characteristics in terms of current level and threshold voltage. The field-effect mobilities of Type A and Type B devices were calculated from the transfer characteristic curves and found to be 0.175 ± 0.01 and 0.185 ± 0.02 cm^2^/Vs, respectively. Note that the measured electrical values such as field-effect mobility and threshold voltage are obtained at least 15 samples.

**Figure 3:**
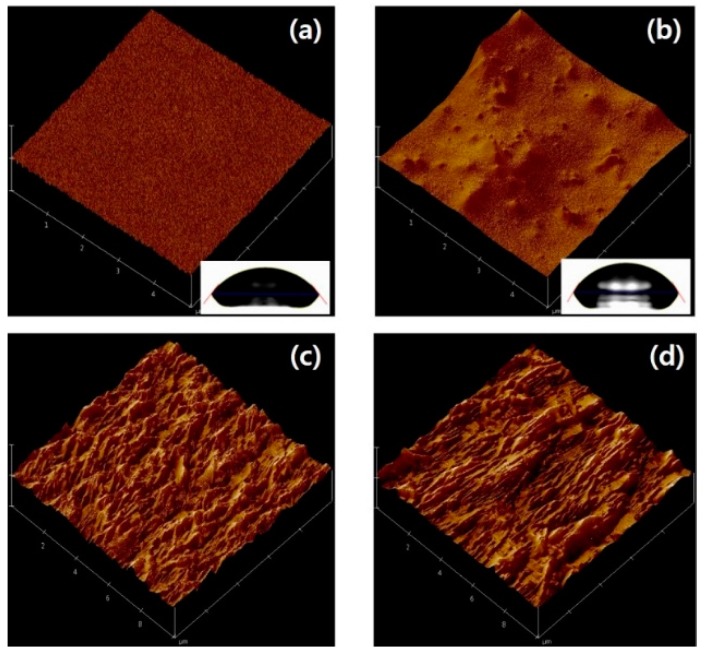
AFM images of the (**a**) c-PVP and (**b**) c-PVP/Y_2_O_3_ composite films. The insets show the contact angles on both films. AFM images of the TIPS-pentacene films deposited on the (**c**) c-PVP and (**d**) c-PVP/Y_2_O_3_ composite films.

The surface properties of c-PVP and c-PVP/Y_2_O_3_ composite films were observed because the surface characteristics of the gate insulator intimately affect the electrical stability of organic TFTs. The AFM images in Figure 3a,b showed that the c-PVP/Y_2_O_3_ nanocomposite film had a rough surface with a root-mean-square (RMS) roughness value of 11.3 nm, whereas the surface of the c-PVP film was much smoother and had an RMS roughness value of 0.35 nm. However, the contact angle of ~54.2° of a distilled-water drop on the c-PVP film was comparable to that the contact angle of ~56.3° on the c-PVP/Y_2_O_3_ film, as shown in the insets of Figure 3a,b. The surface energy of each insulator (*γ_P_*) can be evaluated by the following equation:

(1) $$\gamma_{P}=\frac{\gamma_{W}}{4}{\left(1+\text{cos}\theta_{0}\right)}^{2}$$

where *γ_W_* is the surface free energy of water (73.0 mJ/m^2^), and *θ*_0_ is the measured contact angle at equilibrium [15]. In our results, the c-PVP and c-PVP/Y_2_O_3_ composite films exhibited surface energies of 45.8 and 44.1 mJ/m^2^, respectively. Accordingly, the most distinctive difference in the surface properties of the c-PVP and the c-PVP/Y_2_O_3_ composite films was found to be the surface roughness. This is an important observation to keep in mind when investigating the electrical and mechanical stabilities of fabricated TFTs because the rough surface of the c-PVP/Y_2_O_3_ composite insulator affects both the charge transport behavior at the conducting channel of TFTs and the physical adhesion characteristics between the composite insulator and the TIPS-pentacene semiconductor layers. On the other hand, there was no meaningful difference in the surface morphologies of the TIPS-pentacene films deposited on the c-PVP and c-PVP/Y_2_O_3_ composite insulators, as shown in Figure 3c,d.

**Figure 4:**
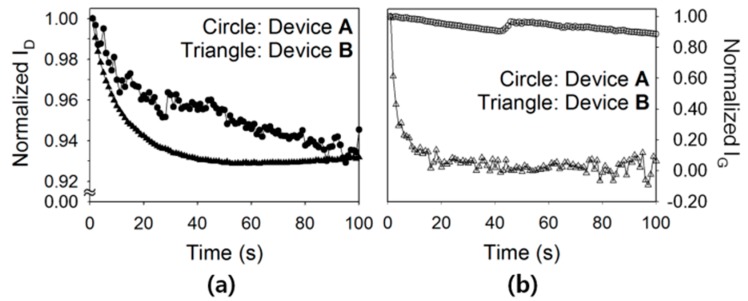
Time-dependent decay behaviors of the (**a**) drain and (**b**) gate currents of the flexible TIPS-pentacene TFTs, during a prolonged bias stress test.

**Figure 5:**
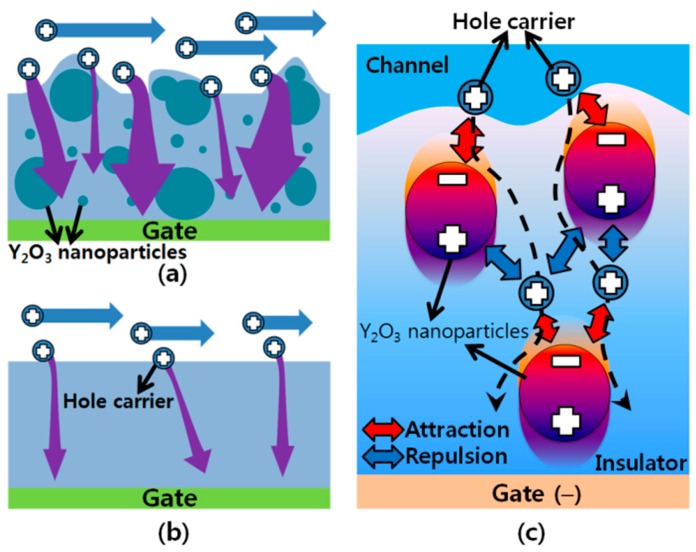
Leakage current paths through the (**a**) c-PVP/Y_2_O_3_ composite and (**b**) c-PVP gate insulators. (**c**) Possible interaction between the holes and the Y_2_O_3_ nanoparticles in the c-PVP/Y_2_O_3_ composite insulator.

To evaluate the electrical stability of Type A and Type B devices, the variations in drain and gate currents during a prolonged bias stress were examined. First, the drain and gate currents were measured in ambient air while a constant drain and gate voltage of −40 V was applied for 100 s. The values of the measured drain and gate current were normalized based on the values obtained at Time = 1 s, and the results are presented in Figure 4. In terms of the variation in drain current, a gradual decrease was observed in both Type A and Type B devices, as shown in Figure 4a. This gradual decrease is thought to be correlated with the charge trapping phenomena in and around the channel region formed at the semiconductor/insulator interface. Indeed, the conducting charge carriers (*i.e.*, holes) in the channel of the TIPS-pentacene TFTs can be trapped at grain boundaries in the TIPS-pentacene layer. The surface energy and surface roughness of the polymeric insulator can also affect the charge transport at the insulator/semiconductor interface [16]. In our results, during the bias stress test for 100 s, both TFTs exhibited a similar change in drain current of approximately 7%. Meanwhile, a rather slow decay was observed for Device A, suggesting that the rough surface of the composite insulator interfered with hole conduction at the interface between the c-PVP/Y_2_O_3_ composite insulator and the TIPS-pentacene semiconductor. In terms of the variation in gate current, a decrease of only ~10% was observed in Type A device, whereas the normalized gate current approached zero in Type B device, as shown in Figure 4b. The reduction in gate current that occurred in both Type A and Type B devices was due to the charge trapping at the semiconductor/insulator interface and the interior of the insulator. With regard to the slowly decreasing gate current, Type A device had more leakage paths for gate current within its nanocomposite insulator than did Type B device, thereby resulting in a gate current that decreased at a lower rate, as shown in Figure 5a,b. One possible explanation of the additional leakage paths in Type A device is as follows. First, hole charge carriers are injected from the channel into the insulator and attracted by the highly polarized nanoparticles. Second, the attracted carriers are released to flow along the direction of the gate electric field. Third, the carriers are repelled by the positive side of the nanoparticles, and attracted by other adjacent nanoparticles, as shown in Figure 5c.

**Figure 6:**
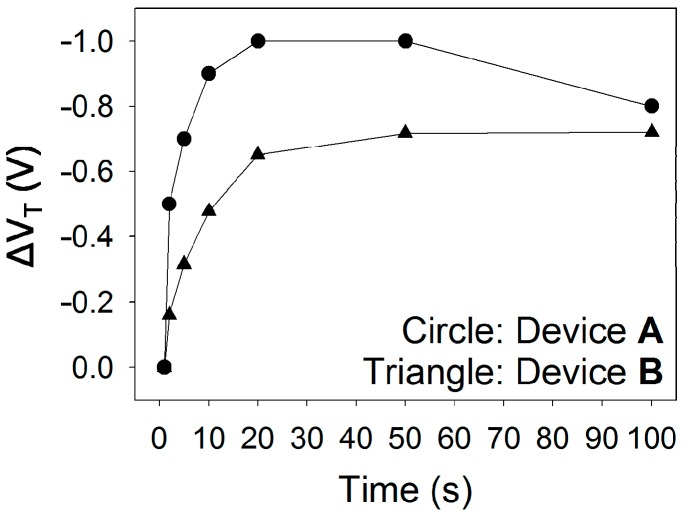
Variations in the threshold voltages of the flexible TIPS-pentacene TFTs with the c-PVP/Y_2_O_3_ composite and c-PVP gate insulators, during a prolonged bias stress test.

In addition to the examination of the drain and gate currents, the variation in threshold voltage during a prolonged bias stress test was also examined in order to evaluate the electrical stability. A constant gate voltage of −10 V was applied to the devices, and the transfer characteristics were measured in order to determine the variation in threshold voltage. The threshold voltage was extracted from the linear regression of the square-root of drain current *vs.* gate voltage curve. Figure 6 shows the change in the threshold voltage (Δ*V*_T_) based on the stress time. In both Type A and Type B devices, the threshold voltage was displaced in the negative direction as the stress time increased until it became saturated. The gradual negative shift in the threshold voltage was due to an increase in the charges trapped at or around the semiconductor/insulator interface which impeded the formation of channel, and the saturation behavior presumably occurred when the trapping sites were fully occupied by charges. Although the threshold voltage of Type A device increased with a slightly higher rate as compared to that of Type B device, the difference in the bias instability of the threshold voltage was comparable between Type A and Type B devices. Based on the results of the electrical stability experiments, it was shown that the detrimental effect of the Y_2_O_3_ nanocomposite insulator on the electrical stability of the device was not critical. Note that the electrical stability of organic TFT using PVP as a gate insulator is closely related to the surface energy of the gate insulator, which is affected by the existence of surface hydroxyl groups [17]. There is no significant difference between the surface energy of the Y_2_O_3_ nanocomposite insulator and that of the native c-PVP insulator, which led to the comparable electrical stability in Type A and Type B devices. However, the gate current, which tends to maintain its initial level during a prolonged bias stress test, would become problematic for integrated circuits.

**Figure 7:**
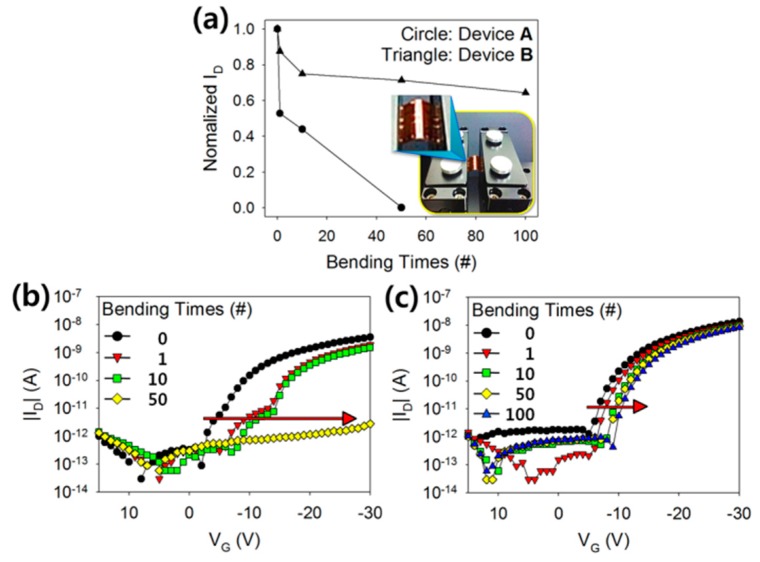
(**a**) Variations in the drain current of the fabricated TFTs, induced by cyclic bending stresses. Transfer characteristics of the flexible TIPS-pentacene TFTs with the (**b**) c-PVP/Y_2_O_3_ composite and (**c**) c-PVP gate insulators, measured during cyclic bending stress tests.

To evaluate the mechanical stability of Type A and Type B devices, the variation in the transfer characteristic under cyclic bending stresses was examined. It was assumed that the TIPS-pentacene and polymeric insulator layers undergo a similar degree of mechanical strain because the total thickness of TIPS-pentacene and polymeric insulator layers (<2 μm) is much thinner than that of the PI substrate (~200 μm). In our study, the flexible substrate was bent with a bending radius (*R*_C_) of 10.2 mm in order to impose mechanical stresses on the TIPS-pentacene TFTs. The amount of strain applied to the TFTs was 1.25 %, which was calculated using the following equation,

(2) $$Strain(\%)=\frac{t_{layer}+t_{substrate}}{2\times R_{C}}$$

where *t_layer_* is the total thickness of the polymeric insulator and the TIPS-pentacene semiconductor layers, and *t_substrate_* is the thickness of the PI substrate [18]. A cyclic bending test was performed at a speed of 800 mm/min. After applying mechanical stresses through the cyclic bending of the fabricated devices, the transfer characteristics were measured with the drain voltage fixed at −30 V and a gate voltage that was swept from 15 to −30 V in −1 V increments. The bending operations were performed in groups consisting of 0, 1, 10, 50, and 100 bends in sequence. Figure 7a shows the variation in the drain current at the gate voltage of −30 V according to the number of bending times. In order to normalize the drain current, the values of drain current were divided by their initial values prior to the bending operations. The drain current decreased when the number of bending times was increased for both Type A and Type B devices, and the decrease was due to the cracks that developed in the TIPS-pentacene film. When compared to Type B device, the normalized drain current decreased more drastically in Type A device, and no longer exhibited any of the electrical characteristics of a transistor after undergoing 100 times bending operation. In the case of Type A device, the rough surface of the nanocomposite insulator caused a non-uniform distribution of bending stresses at the interface to the TIPS-pentacene layer, which presumably induced a detrimental effect on the physical adhesion between TIPS-pentacene molecules and the c-PVP/Y_2_O_3_ nanocomposite insulator. Consequently, severe cracks developed in the TIPS-pentacene film due to the fragile adhesion of the TIPS-pentacene molecules. Figure 7b,c show the displacement of the transfer characteristic curve as well as the decrease in drain current induced by the bending operation. The transfer characteristic curves gradually shifted in the negative direction when the number of bending times was increased for both Type A and Type B devices. This was attributed to the charge accumulation and transport that was impeded by the development of cracks in the TIPS-pentacene film. It is important to note that the displacement tendency was shown to be more severe in Type A device than Type B device. The rapid degradation of Type A device was attributed to the development of severe cracks in the TIPS-pentacene film where it interfaced with the rough surface of the nanocomposite insulator. Considering that the appearance of cracks in TIPS-pentacene depends inevitably on the degree of strain induced in the flexible substrate, the quantitative analysis on the mechanical stability of flexible TFTs by varying the strain condition is required for further investigations. Based on these results, the morphological properties of nanocomposite insulators were shown to be critical factors in terms of the mechanical stability of organic TFTs. The modification of the rough surface of nanocomposite insulators will be an important consideration for flexible low-voltage organic TFTs.

## 4. Conclusions

In this study, flexible TIPS-pentacene TFTs were fabricated using c-PVP/Y_2_O_3_ nanocomposite films as gate insulators, and their electrical and mechanical stabilities were investigated. The devices that were fabricated using nanocomposite or native insulators were shown to have comparable electrical stability by examining variations in their drain current and threshold voltage under gate bias stress. The gate leakage currents showed different tendencies between the two types of devices. The variations in the gate current in the devices with the nanocomposite insulator were explained by suggesting a process where the gate current paths are formed. In terms of mechanical stability experiments, the nanocomposite insulator was shown to have a detrimental effect on the mechanical stability of flexible TIPS-pentacene TFTs when subjected to a repetitive bending operation. In contrast to the case of the native insulator, the transfer characteristic degraded more rapidly as the number of bending times increased in the case of nanocomposite insulator, and this was ascribed to the development of severe cracks in the TIPS-pentacene film where it interfaced with the rough surface of nanocomposite insulator. Our results will be a useful base for the development of flexible electronic devices.
